# Supplementary material for: Inhibition of the RUNX1-CBFβ transcription factor complex compromises mammary epithelial cell identity: a phenotype potentially stabilized by mitotic gene bookmarking
Source: Oncotarget. 2020 Jun 30;11(26):2512–30. doi: 10.18632/oncotarget.27637 (PMC7335667; doi:10.18632/oncotarget.27637)
Supplement: Supplementary file 1 [file oncotarget-11-2512-s001.pdf]

Inhibition of the RUNX1-CBFβ transcription factor complex compromises mammary epithelial cell identity: a phenotype potentially stabilized by mitotic gene bookmarking

SUPPLEMENTARY MATERIALS

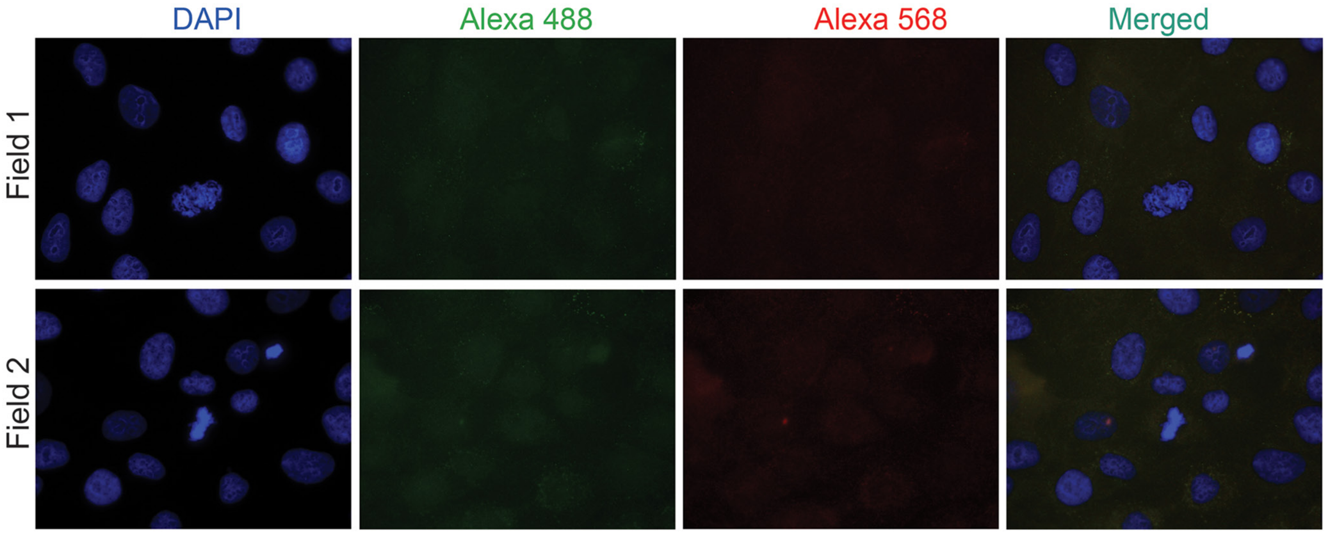

Supplementary Figure 1: Secondary antibody controls for immunofluorescence microscopy. To ensure the specificity of RUNX1 signal on mitotic chromosomes, actively proliferating mammary epithelial MCF10A cells, grown on gelatin-coated coverslips, were subjected to immunofluorescence microscopy procedure as described in Materials and Methods section with one modification: no primary antibody was added, but secondary antibodies were used at the same dilution as in all IF experiments. Nuclei were counterstained with DAPI. Two different fields are shown, confirming that the RUNX1 signal we observe on mitotic chromosomes is specific.

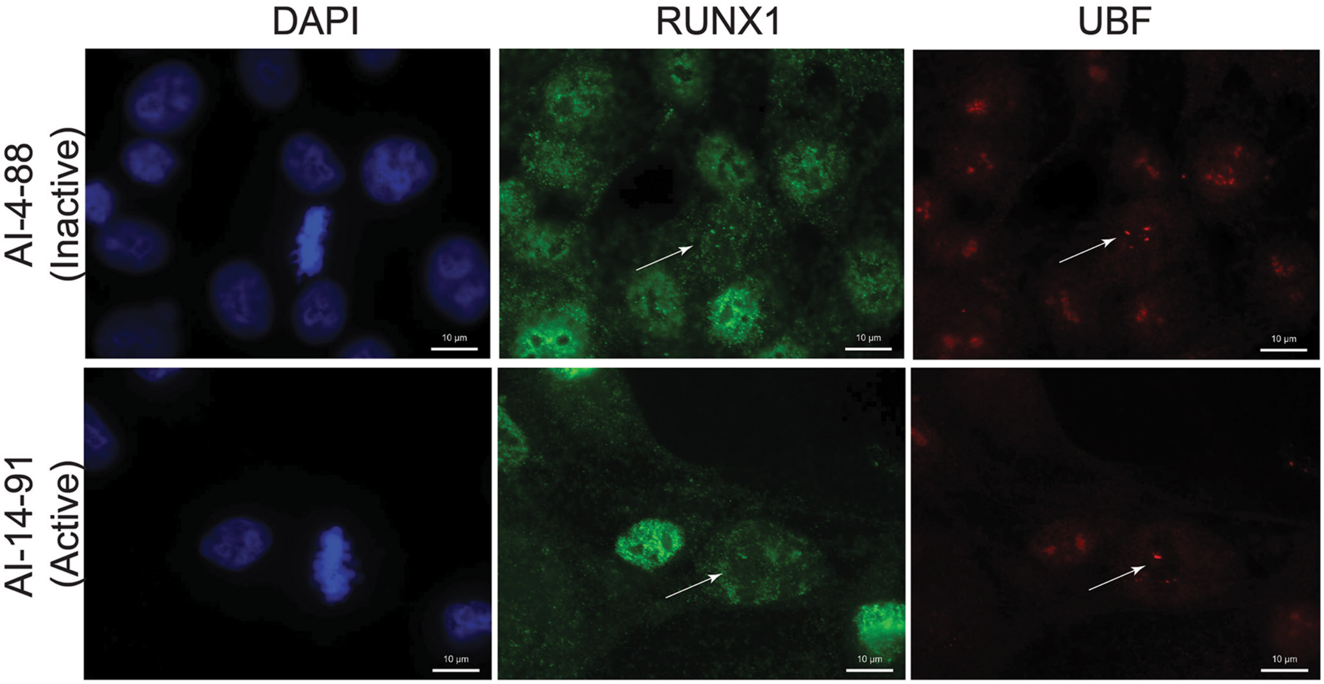

Supplementary Figure 2: The RUNX1-CBFβ inhibitor reduces RUNX1 association with mitotic chromosomes. MCF10A cells, treated with 20 µM inactive control compound or active inhibitor for 12 hours, were stained for localization of endogenous RUNX1 (green), UBF (red) to mitotic chromosomes (DNA; blue). RUNX1 retention on mitotic chromosomes, particularly in smaller foci, was substantially reduced in cells treated with active inhibitor of RUNX1-CBFβ interaction, which disrupts RUNX1 DNA binding activity.

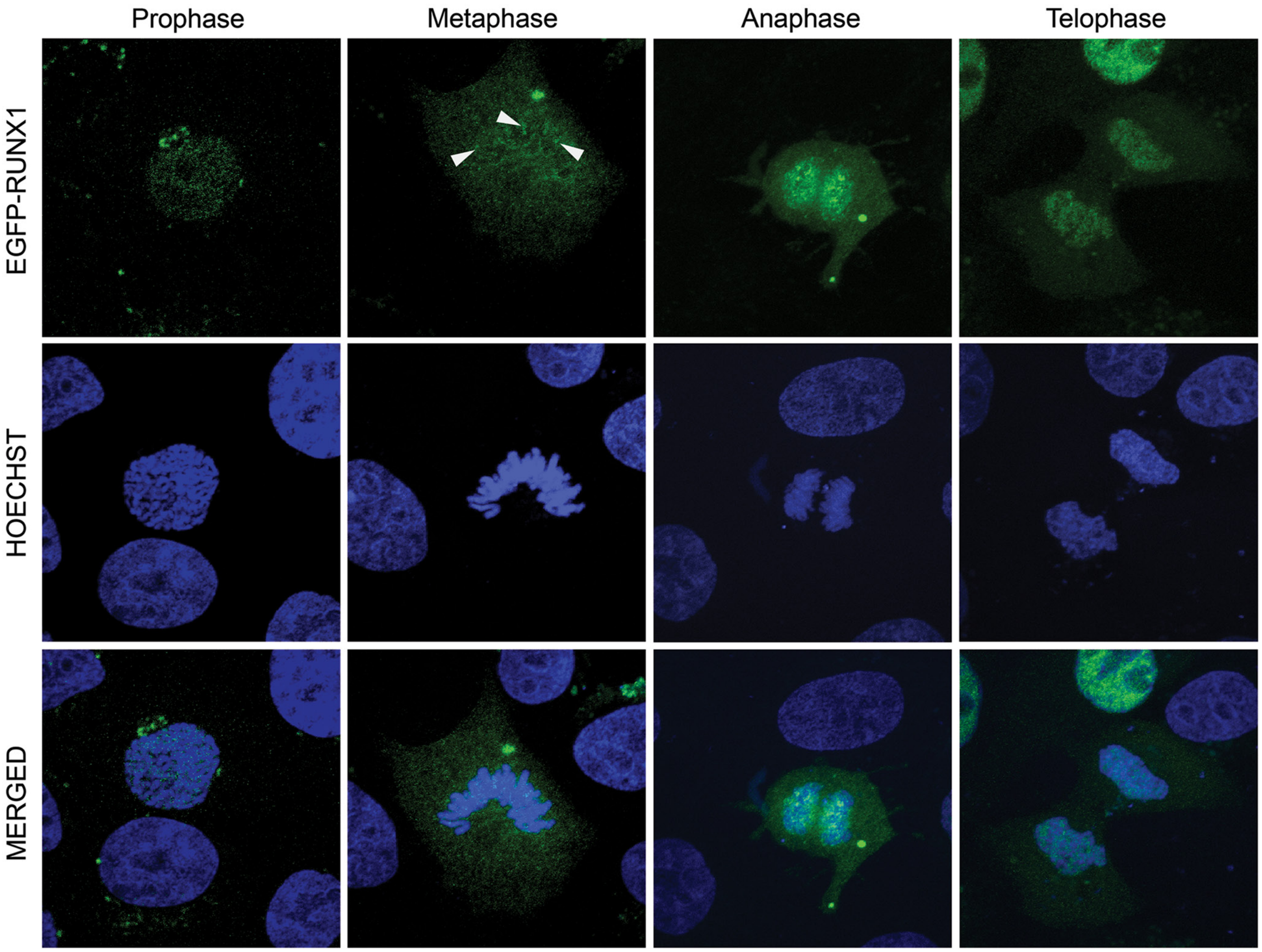

Supplementary Figure 3: RUNX1 associates with chromosomes during all stages of mitosis in unfixed live MCF10A cells. Mammary epithelial MCF10A cells were transiently transfected with EGFP-RUNX1 and imaged by confocal microscopy without fixation (see Materials and Methods section for details). Top panels show RUNX1 (green) association with mitotic chromosomes in unfixed, live MCF10A cells. Cells were counterstained with Hoechst (middle panels; blue) to visualize DNA in live cells and to identify mitotic cells. Merged images (bottom panels) were generated to confirm localization of RUNX1 signal with DNA. Arrow heads indicate punctate RUNX1 foci retained on mitotic chromosomes.

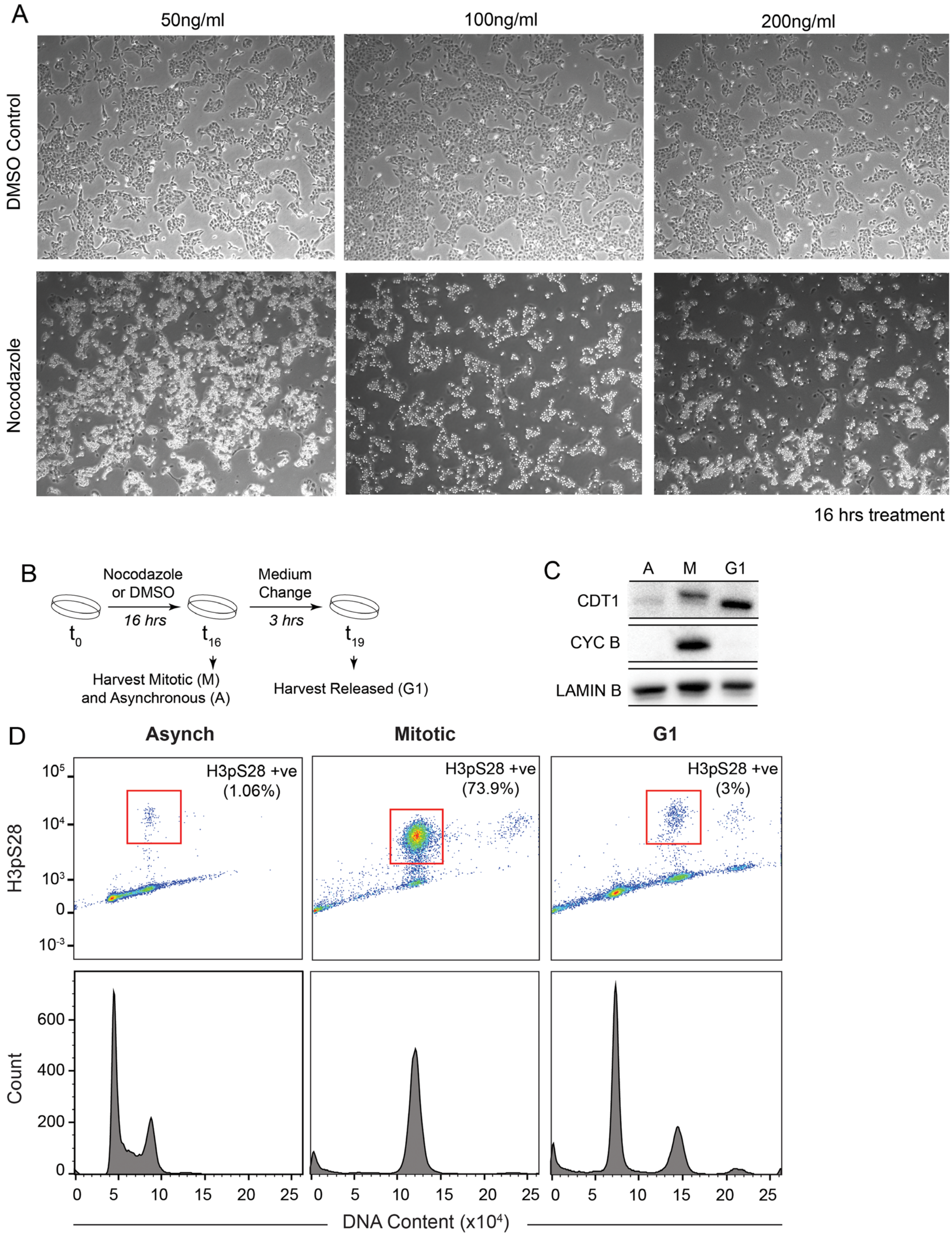

Supplementary Figure 4: Determination of optimal nocodazole dosage for maximum mitotic block. (**A**) Micrographs of MCF10A cells, treated with various doses of nocdazole for 16 hours, are shown (bottom panels). DMSO control treatment is also included (top panels). (**B**) Experimental schematic depicting mitotic arrest and harvest of each treated MCF10A cell population: Asynchronous – A, Mitotic – M, and Released – G1. (**C**) Western blot of each harvested MCF10A population for cell cycle specific markers to evaluate mitotic arrest and synchronization procedure. (**D**) Fluorescence-activated cell sorting (FACS) analysis of harvested A, M, and G1 MCF10A populations to determine mitotic purity and DNA content (n = 2 biological replicates per group).

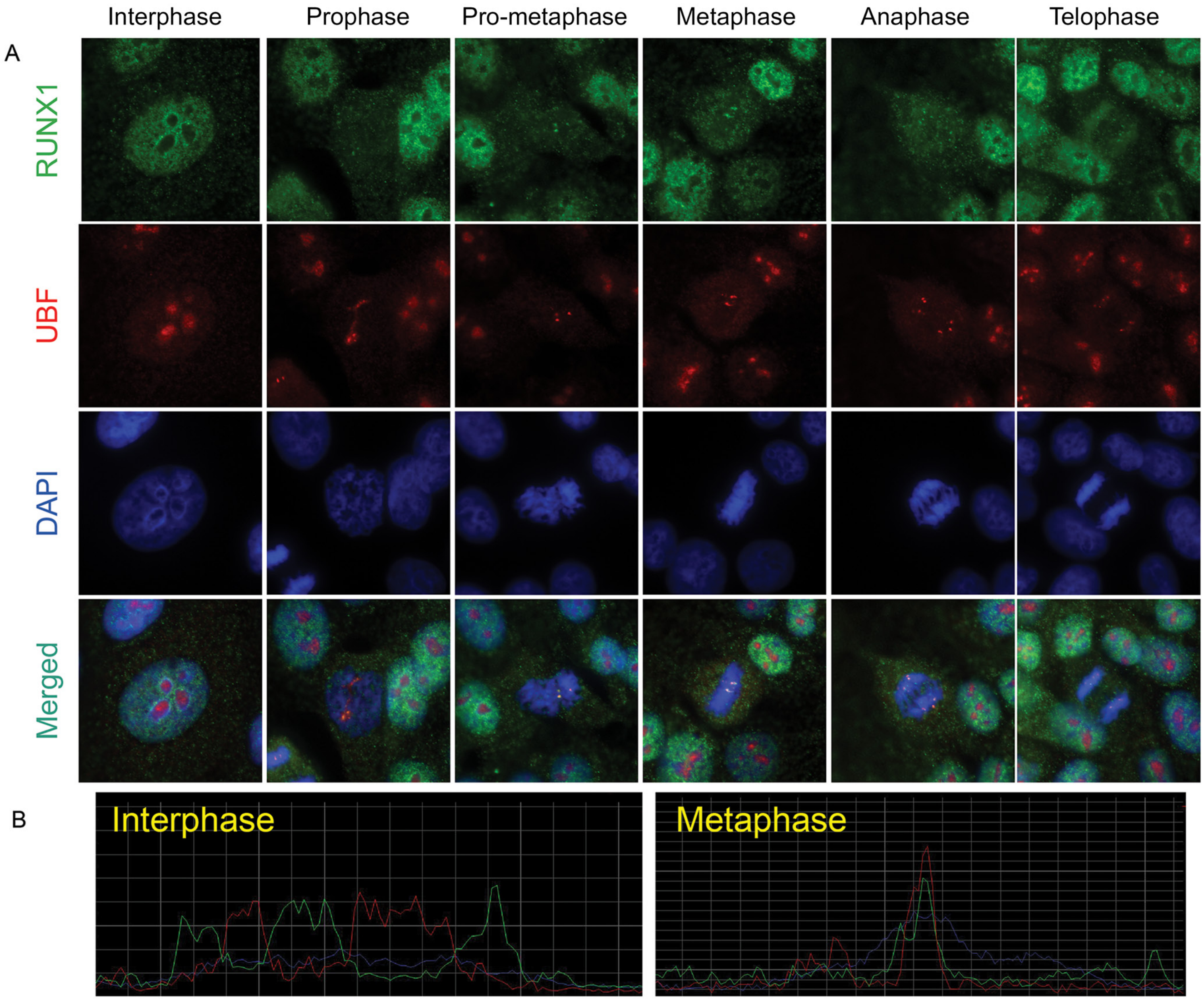

Supplementary Figure 5: RUNX1 colocalizes with RNA Pol I subunit, upstream binding factor (UBF) on mitotic chromatin. (**A**) Immunofluorescence microscopy images of RUNX1 (green – top row), UBF (red – 2nd row from top), DAPI (blue – 2nd row from bottom), and the three channels merged (bottom row) in MCF10A cells. Images were captured of spontaneously dividing MCF10A cells in different substages of mitosis. (**B**) Representative images of line profiles taken on interphase vs metaphase cells (*n* = 15 each).

Supplementary Table 1: Gene set enrichment analysis of genes sensitive to RUNX1-CBFβ inhibition during crisis and recovery phases of epithelial to mesenchymal transition in MCF10A cells

GSEA Report for Crisis

NAME	SIZE	ES	NES	NOM *p*-val	FDR *q*-val	FWER *p*-val	RANK AT MAX
HALLMARK_HEME_METABOLISM	18	0.3947277	1.1353589	0.25240847	1	0.622	708
HALLMARK_MTORC1_SIGNALING	35	0.5021444	1.099158	0.37525773	1	0.696	763
HALLMARK_HYPOXIA	53	0.48392004	1.0695419	0.31150794	1	0.717	884
HALLMARK_GLYCOLYSIS	32	0.39393955	1.006081	0.42460316	1	0.781	793
HALLMARK_UNFOLDED_PROTEIN_RESPONSE	16	0.5152979	0.9632453	0.511811	1	0.792	918
HALLMARK_TNFA_SIGNALING_VIA_NFKB	69	0.4499188	0.959573	0.54789275	1	0.803	975
HALLMARK_INFLAMMATORY_RESPONSE	32	0.38814172	0.92550886	0.45388788	1	0.814	975
HALLMARK_INTERFERON_GAMMA_RESPONSE	40	0.41000193	0.8577103	0.6434783	1	0.831	949
HALLMARK_UV_RESPONSE_UP	22	0.4523549	0.843655	0.6709129	1	0.846	939
HALLMARK_IL2_STAT5_SIGNALING	27	0.35796466	0.8086394	0.66386557	1	0.872	940
HALLMARK_KRAS_SIGNALING_UP	31	0.372217	0.8083701	0.64299804	0.9587773	0.872	967
HALLMARK_INTERFERON_ALPHA_RESPONSE	17	0.3713511	0.77638674	0.66267467	0.95283103	0.884	876
HALLMARK_COMPLEMENT	21	0.30336428	0.6690581	0.74251497	1	0.907	961
HALLMARK_XENOBIOTIC_METABOLISM	20	0.24546017	0.62348866	0.8779599	1	0.919	787
HALLMARK_APOPTOSIS	30	0.26021215	0.60873795	0.6981132	1	0.919	958
HALLMARK_EPITHELIAL_MESENCHYMAL_TRANSITION	28	0.22567452	0.60038143	0.90679306	0.9498569	0.919	950
HALLMARK_P53_PATHWAY	37	0.20076397	0.5406085	0.94708997	0.9333686	0.919	636
HALLMARK_APICAL_JUNCTION	24	–0.1968617	–0.5646497	0.9259259	0.9119534	0.882	67
HALLMARK_ESTROGEN_RESPONSE_EARLY	36	–0.2092965	–0.6075348	0.8805195	1	0.862	406
HALLMARK_UV_RESPONSE_DN	15	–0.3342217	–0.9270731	0.5578704	0.7091259	0.8	187
HALLMARK_ESTROGEN_RESPONSE_LATE	30	–0.3937942	–1.0697397	0.41567695	0.63777864	0.666	318
HALLMARK_ADIPOGENESIS	21	–0.4425912	–1.262755	0.1050505	0.4333697	0.472	317
HALLMARK_KRAS_SIGNALING_DN	17	–0.6807129	–1.3786752	0.07142858	0.42511922	0.311	321

GSEA Report for Recovery

NAME	SIZE	ES	NES	NOM *p*-val	FDR *q*-val	FWER *p*-val	RANK AT MAX
HALLMARK_HYPOXIA	15	0.41979817	0.9262716	0.5611888	1	0.57	507
HALLMARK_INTERFERON_GAMMA_RESPONSE	21	0.41107994	0.8376136	0.741533	1	0.658	519
HALLMARK_EPITHELIAL_MESENCHYMAL_TRANSITION	15	0.39494085	0.75395024	0.63284135	1	0.686	652
HALLMARK_COMPLEMENT	19	0.35864845	0.8134814	0.6788194	1	0.686	657
HALLMARK_TNFA_SIGNALING_VIA_NFKB	27	0.34575072	0.73579097	0.83908045	0.88889724	0.7	700
HALLMARK_KRAS_SIGNALING_UP	22	0.20223154	0.5116736	0.98634297	0.9498962	0.807	700

Supplementary Table 2: List of genes occupied by RUNX1 in asynchronous, mitotic and G1 cell populations in mammary epithelial cells

ZNF839	Cluster4
ARL6IP1	C16orf72
AZIN2	C8orf37
BTRC	CBWD5
CARNMT1	CCDC114
CCDC130	CCDC50
CD55	CYTH2
CLEC16A	DECR1
COL9A1	DOHH
CYP51A1-AS1	DUSP1
DUSP19	ELOBP4
EGR3	FABP6

EXD3	FAM129B
EXOSC4	FAM225B
FAM8A1	GGT7
FSCN1	GPR39
FTSJ1	GSDME
GNL3	GSE1
GSPT1	GZF1
GTF2A2	HCCS
H2AFX	HIPK2
HYLS1	LAMB2
IER3-AS1	LINC02227
KDM7A	LINC02580
KLK10	MAF1
LINC01004	MAL2-AS1
LINC01804	MIR3922
LRRN3	MPLKIP
MCC	MRPL38
METTL27	NAPA
MIR661	NBPF19
MYEOV	NCF1C
MYL12A	PIGK
PCDH1	PRSS16
RN7SL189P	PSIP1
RNF139-AS1	RAD9A
RNPEPL1	RN7SL76P
RNU6-1264P	RNA5SP158
TGFB2	RNA5SP220
TPRG1-AS2	RSPH6A
TRIP11	RTEL1
UBE2T	SGO1
ZG16B	SLC27A5
ZNF181	SNRPA
ZNF302	SNX16
	SRGAP3
	TCEA3
	TPR
	USP3-AS1
	WISP2
	XDH
	ZKSCAN3
	ZNF584
	ZNF839
